# Evaluation of Qatar’s First Cardiac Rehabilitation Program: A Brief Report

**DOI:** 10.5334/gh.862

**Published:** 2021-10-08

**Authors:** Eman Faisal, Rahma Saad, Mohammed Al-Hashemi, Sherry L. Grace, Theodoros Papasavvas, Karam Turk-Adawi

**Affiliations:** 1 Qatar University, QU Health, Department of Public Health, Doha, QA; 2 Hamad Medical Cooperation, Heart Hospital, Doha, QA; 3 York University & KITE-Toronto Rehabilitation Institute, University Health Network, University of Toronto, CA

**Keywords:** Cardiac rehabilitation, health-related quality of life, cardiovascular disease, adherence, quality of care, depression

## Abstract

**Background:**

There are few studies on the impact of cardiac rehabilitation (CR) in the Eastern Mediterranean Region (EMR), where the burden of risk factors and context is somewhat different from Western countries where much of the evidence is derived.

**Objective:**

To evaluate patient engagement in, and outcomes associated with, participation in Qatar’s first and only CR program, from inception.

**Methods:**

This was a retrospective, observational study of patients referred to Heart Hospital’s CR program from January 2013-September, 2018. The program offered 3 sessions/week over 6–12 weeks, depending on patient risk. An initial assessment was performed, and outcomes (i.e., functional capacity, risk factors, and psychosocial well-being (quality of life [SF-36] and depressive symptoms) were re-assessed post-program in those who did not drop-out. Session attendance was recorded.

**Results:**

682 patients enrolled; they attended 77.6% of prescribed sessions; 554 (81.2%) completed the program and post-assessment. Improvements in functional capacity were statistically and clinically meaningful (METs 9.3 ± 3.3 pre and 11.1 ± 3.7 post; p < 0.001). There were significant improvements in body mass index (28.7 ± 5.2 kg/m^2^ pre and 28.2 ± 5.4 post; p < 0.001), waist circumference (102.8 ± 13.0 cm pre and 101.8 ± 13.2 post; p < 0.001), low-density lipoprotein (LDL 1.9 ± 0.9 mmol/L pre and 1.6 ± 0.8 post; p = < 0.001), total cholesterol (3.6 ± 1.1 mmol/L pre and 3.3 ± 0.8 post; p < 0.001), systolic blood pressure (SBP 128.5 ± 17.7 mmHg pre and 123.7 ± 14.8 post; p < 0.001), hemoglobin A1c (6.8 ± 1.6% pre and 6.5 ± 1.3 post; p < 0.001) and depressive symptoms (Cardiac Depression Scale score 78.3 ± 23.9 pre and 66.3 ± 21.3 post; p < 0.001). Improvements on 7 of the 8 quality of life domains were also observed (all p < .05; e.g., physical functioning 68.2 ± 24.0 pre and 74.9 ± 24.4 post).

**Conclusion:**

The new Qatari CR program is very engaging to patients, and resulted in clinically significant risk factors (LDL, SBP, and cholesterol) as well as functional capacity and health-related quality of life improvements, which likely translate to reduced morbidity and mortality.

Cardiovascular diseases (CVD) in the Eastern Mediterranean Region (EMR) are the leading cause of disability, accounting for 9.2% of total disability-adjusted life years. The burden of risk factors is also very high in those with CVD in the EMR, yet there is poor control. This puts patients at increased risk of recurrent events.

Cardiovascular rehabilitation (CR) is an established model of outpatient care to mitigate this burden. Network meta-analyses of randomized trials, all of them primarily from Western settings, demonstrate that CR participation results in significantly lower mortality and morbidity. However, despite the fact that CR exists in 12 of the 22 EMR countries [1], there have been few studies on the benefits of CR in the EMR, stemming only from Saudi Arabia, Egypt, Pakistan and Iran [2,3,4,5]. This is despite the fact that risk factor burden (e.g., tobacco use) and hence patient profile differs in this context, which could impact program utilization and outcomes. Indeed, there are also differences in culture, transport, weather and environment, among other factors, in the EMR compared to other regions which could impact utilization, and hence outcomes.

In 2013, Qatar opened a first CR program; it remains the sole program in the country. The purpose of the current study was to evaluate patient: (1) engagement in the program (i.e., adherence and completion), and (2) change in outcomes (i.e., functional capacity, risk factors and psychosocial well-being (quality of life [QoL] and depressive symptoms)) from pre- to post-program among those participating in this new program for the first time.

This is an observational retrospective cohort study, analyzing patients’ records obtained from the CR program at the Heart Hospital in Qatar. A waiver of patient consent was received (Hamad Medical Cooperation Medical Research Center [MRC-01-18-431], and Qatar University Institutional Review Board [QU-IRB 1068-E/19]).

Details about the institutional setting and CR program are shown in the Supplemental file.

The study sample included all patients who enrolled in the sole Qatar CR program, from program inception (January 2013) to September 2018. Patients were ≥18 years. There were no exclusion criteria for the study.

For objective 1, adherence was defined as proportion of prescribed sessions attended. Completion was defined as undertaking the post-program assessments. Outcomes were: functional capacity (peak metabolic equivalents [METs]), risk factors (i.e., systolic blood pressure [SBP; mmHg] using automated blood pressure monitors, lipids [cholesterol, low-density lipoprotein (LDL), high-density lipoprotein (HDL)], body mass index [BMI; kg/m^2^], and self-reported tobacco use), as well as psychosocial well-being (QoL [SF-36] and depressive symptoms [Cardiac Depression Scale]; further details regarding these scales are found in the Supplemental file).

Data were analyzed using STATA v16. A paired-sample t-test was performed to assess change from pre- to post-program in the continuous outcome measures, and chi-square test for categorical measures (e.g., tobacco use % pre to post-program).

During the period of study, 682 patients enrolled in the program; their characteristics are shown in Supplemental Table 1. Participants attended a mean of 20.4 ±12.3 sessions (median = 22.0), or 77.6% of prescribed sessions and 554 (81.2%) patients completed the program and post-program assessment. As also shown in the Table, there was some retention bias.

As shown in Table 1, participants experienced statistically-significant improvements in all outcomes, except for HDL. QoL also improved significantly in all domains except pain. There was a statistically significant reduction in the proportion of current tobacco users from pre to post-program.

**Table 1:** Change in Patient Outcomes, N = 554.

Outcome	Pre-CR	Post-CR	Mean Difference	95% CI		p

Functional capacity (METs)	9.3 ± 3.3	11.1 ± 3.7	1.8 ± 2.1	1.63	1.98	<0.001
Risk Factors						
BMI (kg/m^2^)	28.7 ± 5.2	28.2 ± 5.4	–0.5 ± 2.2	–0.70	–0.31	<0.001
WC (cm)	102.9 ± 13.0	101.8 ± 13.2	–1.2	–1.6	–0.6	<0.001
LDL (mmol/L)	1.9 ± 0.9	1.6 ± 0.80	–0.3 ± 0.8	–0.38	–0.24	<0.001
HDL (mmol/L)	1.0 ± 0.3	1.1 ± 0.3	0.0 ± 0.3	–0.00	0.04	0.066
Cholesterol (mmol/L)	3.6 ± 1.1	3.3 ± 0.8	–0.3 ± 0.9	–0.39	–0.24	<0.001
SBP (mmHg)	128.5 ± 17.7	123.7 ± 14.8	–4.7 ± 15.9	–6.08	–3.42	<0.001
HbA1c (%)^‡^	6.8 ± 1.6	6.5 ± 1.3	–0.3 ± 15.9	–0.45	–0.22	<0.001
Current tobacco use (%)	107 (19.3)	50 (9.0)	–10.3^†^			<0.001
Quality of life*:						
Physical functioning	68.2 ± 24.0	74.9 ± 24.4	6.7 ± 25.9	2.08	11.35	0.004
Social functioning	77.9 ± 22.2	84.5 ± 18.1	6.5 ± 22.3	2.66	10.53	0.001
Emotional well-being	75.5 ± 17.5	81.3 ± 15.3	5.8 ± 15.9	3.13	8.57	<0.001
General health	67.8 ± 19.2	74.6 ± 19.1	6.8 ± 15.9	4.10	9.61	<0.001
Role limitation due to physical health	71.1 ± 36.3	81.9 ± 30.5	10.0 ± 34.7	3.85	16.31	0.002
Role limitation due to emotional problems	74.1 ± 37.0	81.1 ± 32.3	7.0 ± 38.1	0.18	13.86	0.044
Energy/fatigue	67.6 ± 18.6	74.1 ± 17.0	6.5 ± 16.1	3.57	9.42	<0.001
Pain	79.0 ± 22.7	82.1 ± 18.9	3.1 ± 21.2	–0.74	6.94	0.113
CDS (scores)	78.3 ± 23.9	66.3 ± 21.3	–12.0 ± 15.3	–13.3	–10.8	<0.001

Values are expressed as mean ± standard deviation. CI, confidence interval; CR, cardiac rehabilitation; BMI, body mass index; WC, waist circumference; LDL, low-density lipoprotein; HDL, high-density lipoprotein; SBP, systolic blood pressure; HbA1c, hemoglobin A1c; CDS, Cardiac Depression Scale (CDS). * n = 124. According to Cohen, an effect size of 0.2 is an indicator of at least a small effect [9]. Effect sizes for physical functioning (d = 0.27, 95% confidence interval [CI] = 0.11–0.47), social functioning (d = 0.29, 95% CI = 0.11–0.47), emotional well-being (d = 0.33, 95% CI = 0.17–0.48), role limitation due to physical health (d = 0.27, 95% CI = 0.10–0.44), role limitation due to emotional problems (d = 0.21, 95% CI = 0.005–0.42), energy/fatigue (d = 0.34, 95% CI = 0.19–0.50) and general health (d = 0.35, 95% CI = 0.21–0.50) were all above this threshold. ^‡^ Among n =224 patients diagnosed with diabetes. ^†^ Change in percentage.

This is the first study to test the effects of CR in Qatar; as anticipated the patients who accessed CR were very engaged in the program, and realized significant improvements in outcomes; the implications of these findings are shown in the Supplemental Appendix. Although rates of adherence and completion vary and are not well-established, these rates do seem much higher than what is observed in Western settings (e.g., 70% adherence in a meta-analysis) [6], but are consistent with the higher adherence shown in other low-resource settings [7].

Functional capacity improved by 19.3%, or approximately 2 METs, which is greater than the .5–1 MET threshold to indicate a “quality” program and which is associated with mortality reductions [8]. With regard to risk factors, LDL decreased by 15.7%, which could be due to adherence to statins (>90%). A reduction of 10% is clinically significant as it is associated with reduced coronary deaths. Moreover, SBP decreased ≥2 mmHg, considered clinically-significant. BMI decreased by 1.7%, which would not be considered clinically significant; However, this weight loss was statistically significant and often does not reach significance in most studies, so the results of the program are promising. Tobacco use reductions are also often not assessed or observed with CR, so the reduction observed with the Qatar CR program is very promising, given tobacco cessation is the most impactful secondary prevention change in terms of reducing mortality. QoL subscale scores significantly increased in all domains except pain; this is likely because of a ceiling effect. Effect sizes are shown in Table 1, and at least small [9]. Reductions in depressive symptoms were similarly remarkable.

Caution is warranted when interpreting these results. First, with regard to generalizability, data come from a single centre, and therefore generalizability to other programs in the EMR is unknown; certainly, these results are consistent with the benefits observed with CR in Iran [2,3,4,5]. Generalizability may also be limited in that the program did not track some key characteristics of the indicated patients at their institution or beyond, to understand how characteristics of the population being referred and receiving CR is different from the characteristics of the population who should be. Second, there is some retention bias in the sample. Third, due to the nature of the design, causal conclusions cannot be drawn; however, these results are in line with the few randomized trials on CR in countries of the EMR [10] and hence it is likely the effects are robust. Indeed, there was no control group, and in future evaluation it will be important to incorporate such a comparison arm.

In conclusion, the new Qatari CR program is very engaging to patients, and results in clinically significant risk factor reduction, as well as functional capacity and psychosocial well-being improvements, which likely translate to reduced morbidity and mortality. Some areas to potentially improve program processes and quality were identified, which will likely be implemented through the program expansion and piloting of the International Cardiac Rehab Registry (ICRR; https://globalcardiacrehab.com/ICRR-Governance).

## Additional File

The additional file for this article can be found as follows:

10.5334/gh.862.s1 Online supplementary file. This includes: Supplemental Table 1; CR setting description; and Measures description.

## References

[1] Turk-Adawi K, Supervia M, Pesah E, Lopez-Jimenez F, Afaneh J, El-Heneidy A, et al. Availability and delivery of cardiac rehabilitation in the Eastern Mediterranean Region: How does it compare globally? Int J Cardiol. 2019; 285: 147–53. DOI: 10.1016/j.ijcard.2019.02.065 30904282

[2] Khalid Z, Farheen H, Tariq MI, Amjad I. Effectiveness of resistance interval training versus aerobic interval training on peak oxygen uptake in patients with myocardial infarction. J Pak Med Assoc. 2019; 69(8): 1194–8. 31431779

[3] Abolahrari-Shirazi S, Kojuri J, Bagheri Z, Rojhani-Shirazi Z. Efficacy of combined endurance-resistance training versus endurance training in patients with heart failure after percutaneous coronary intervention: A randomized controlled trial. J Res Med Sci. 2018; 23: 12. DOI: 10.4103/jrms.JRMS_743_17 29531564 PMC5842444

[4] Ali Mohammed Hassan NGEN. Efficacy of cardiac rehabilitation after percutaneous coronary intervention. International Journal of PharmTech Research; 2016.

[5] Mutwalli H, Fallows S, Ashmeg A, Abukhudair W, Arnous A, Zamzami M. A controlled trial of home-based cardiac rehabilitation versus usual hospital care in cardiac patients. Journal of the Saudi Heart Association. 2012; 24(4): 286. DOI: 10.1016/j.jsha.2012.06.231

[6] Oosenbrug E, Marinho RP, Zhang J, Marzolini S, Colella TJ, Pakosh M, et al. Sex differences in cardiac rehabilitation adherence: A meta-analysis. The Canadian Journal of Cardiology. 2016; 32(11): 1316–24. DOI: 10.1016/j.cjca.2016.01.036 27129618

[7] Chaves G, Ghisi GLM, Grace SL, Oh P, Ribeiro AL, Britto RR. Effects of comprehensive cardiac rehabilitation on functional capacity in a middle-income country: a randomised controlled trial. Heart (British Cardiac Society). 2019; 105(5): 406–13. 30282639 10.1136/heartjnl-2018-313632

[8] Myers J, Prakash M, Froelicher V, Do D, Partington S, Atwood JE. Exercise capacity and mortality among men referred for exercise testing. New England Journal of Medicine. 2002; 346(11): 793–801. DOI: 10.1056/NEJMoa011858 11893790

[9] Cohen J. Statistical power analysis for the behavioral sciences. Routledge; 1988.

[10] Mamataz T, Uddin J, Ibn Alam S, Taylor R, Pakosh M, Grace SL. Effects of cardiac rehabilitation in low and middle-income countries: A systematic review and meta-analysis of randomized controlled trials. Under review. British Medical Journal; 2021. DOI: 10.1016/j.pcad.2021.07.004 PMC9187522 34271035

