# Supplementary material for: Evaluation of Qatar’s First Cardiac Rehabilitation Program: A Brief Report
Source: Glob Heart. 2021 Oct 8;16(1):65. doi: 10.5334/gh.862 (PMC8499716; doi:10.5334/gh.862)
Supplement: Online supplementary file. — This includes: Supplemental Table 1; CR setting description; and Measures description. [file gh-16-1-862-s1.pdf]

Online supplementary file

Evaluation of Qatar's First Cardiac Rehabilitation Program: a brief report

Eman Faisal[1], MPH, Rahma Saad[1], MPH, Mohammed Al- Hashemi[2], MD, Sherry L. Grace PhD, CRFC[3], Theodoros Papasavvas[2], PhD; Karam Turk-Adawi[1], PhD.

1 QU Health, Qatar University, Department of Public Health, Doha, Qatar.

2 Hamad Medical Cooperation, Heart Hospital, Doha, Qatar.

3 York University & KITE-Toronto Rehabilitation Institute, University Health Network, University of Toronto, Canada

Setting

The program is part of the cardiology department in the Heart Hospital; it is the sole referral hospital in Qatar for cardiovascular procedures (i.e. with advanced technology; 442 percutaneous coronary intervention [PCI] and 154 coronary artery bypass graft [CABG] procedures were performed in the institution [N=596 revascularizations], among only patients enrolled in the program). In the Hospital, systematic discharge order sets are used; patients are automatically referred to Qatar's phase II / outpatient CR program from the inpatient CR unit, where the majority of the CR guideline-indicated patients receive treatment. Patients with cardiac indications recommended in the Canadian and the American CR Guidelines are referred, namely myocardial infarction (MI), PCI, CABG, heart valve intervention, heart failure (HF), and heart or heart/lung transplantation. Patients who cannot undergo a symptom-limited exercise test or a 6-minute walk test are excluded. The program does not accept stable angina patients due to insufficient capacity.

The program serves both Qataris and non-Qataris. Both have free and equal access to health services that are financed from public funds [3]. CR services are hence publicly covered ($0 out of pocket patient cost). The program was developed in accordance with the Canadian and the American CR Guidelines [1, 2]. All referred patients are called and offered place to start the program within approximately five months; the wait list is long due to insufficient capacity of the sole program to treat all indicated patients in Qatar.

The CR program is managed by a cardiologist, who is assisted by a program manager and coordinator. The CR staff includes 2 cardiologists, 10 nurses, 4 dietitians, 3 kinesiologists/ exercise specialists, 2 occupational therapist, 2 physiotherapists, a pharmacist, and an administrative assistant/ secretary. All staff are involved in the initial and final assessments, and enter the relevant patient data in the system for their role. The program offers 18 sessions per week. The program serves approximately 200 patients /year, with 6 patients per session.

All patients undergo a symptom-limited exercise test. Most are conducted on a treadmill (some cycle ergometer) without gas exchange measurements; when the patient is unable to undergo a symptom-limited exercise test, a 6-minute walk test is conducted instead. To increase the capacity of the program in serving more patients, patients are offered sessions based on their risk status (low risk – 18 sessions; moderate risk – 24 sessions; high risk – 36 sessions), 3 times per week.

The program components are assessment (pre and post-program), patient education, management of CVD risk factors, structured exercise, nutrition counseling, tobacco cessation sessions/classes, return-to-work counselling (the latter 2 as applicable). For exercise, the prescription is circuit-based using the following equipment: treadmill, cycle ergometer, arm ergometer, stepper, elliptical machine, rower, and free dumbbells. Exercise intensity is

prescribed based on the maximum heart rate achieved during the symptom-limited exercise test. Patients exercise at moderate intensity (55% - 69% of peak heart rate or 45% - 59% of peak oxygen consumption in case of cardiopulmonary exercise test). The duration of the exercise session is 60 minutes, of which 10 minutes warm-up and 10 minutes cool-down. Patients also do resistance training during the exercise sessions. The program offers female-only exercise sessions.

Measures

The 36-item Short-Form Health Survey (SF-36) is a self-administered, generic, comprehensive and frequently-used instrument to measure physical and mental QoL [4]. It includes eight sub-scales: physical functioning, role limitations due to physical health, role limitations due to emotional problems, energy/fatigue, emotional well-being, social functioning, pain, and general health [5]. The SF-36 is a valid and sensitive instrument, and appropriate to be used among CR patients [6]. Scoring of the SF-36 is done in two steps. Firstly, coded values are given to each response category for all the 36 items; each item is scored between 0 to 100, where 0 represents the poorest health, and 100 represents the best [5]. Secondly, the average of all items for each subscale is calculated, to produce the eight subscale scores [5]. QoL was collected up to 2017 only.

Finally, depressive symptoms were measured through the Cardiac Depression Scale (CDS). This scale is appropriate for CR patients [7, 8]. It consists of 26 items, scored on a 7-point Likert scale ranging from 'strongly disagree' (1) to 'strongly agree' (7). The total CDS score is the sum of all items. Thus, it ranges from 26 to 182, and the higher the CDS score, the more severe the depression. Responses were categorized into 3 categories using cut-off values from the literature [7]: patients with scores less than 90 were considered to have subthreshold depressive symptoms, those with scores from 90 to <100 were considered to have mild-moderate severity depressive symptoms, and those with scores ≥100 considered to have severe depressive symptoms [7].

Implications

There is much development of CR in non-Western settings [9], where CVD rates are exploding. It is important that programs are developed in accordance with guidelines, despite more limited resources (for CR; Qatar is rich in other resources), for instance as suggested in the International Council of Cardiovascular Prevention and Rehabilitation's (ICCPR) consensus statement for low-resource settings [10, 11]. Therefore, it will be important to continue to evaluate outcomes, and even to benchmark against other programs. Indeed, ICCPR is currently developing an International CR Registry (ICRR; https://globalcardiacrehab.com/ICRR-Governance), which will be piloted in Qatar, to enable this. Moreover, our program is in the midst of developing a satellite site to increase our capacity and reach, given in Qatar there is a need for 6800 more spots each year to treat incident ischemic heart disease patients alone [12]. Through the registry, we will be able to test whether we have high-quality care across our program, no matter the location. For instance, we must monitor our wait times [13], and hopefully they can be shortened with the additional satellite capacity.

Through examining the data of the new program, we noted some deficiencies in completeness of data. We secured the paper files of patients, to capture any additional information to reduce this missingness. We have recommended the program ensure regular assessment and patient completion of all QoL measures at pre and post-program. Another key outcome that is missing from our data is return-to-roles (e.g., paid and unpaid work). These will be included in the ICRR. In future, it would also be important to test the impact of the program on morbidity and mortality, as well as cost-effectiveness. Indeed, evaluation is a key "core component" of CR [14].

Supplemental Table 1. Sociodemographic and Clinical Characteristic of Patients Pre-Cardiac Rehabilitation and by Retention Status

**Characteristics**	**Total (N=682)**	**Retained (n=554)**	**Lost to follow-up (n=128)**	**p***
Sociodemographic				
Age (years)	54.05±10.0	52.9±9.9	51.7±10.4	.23
Sex (% male)	613 (89.9)	502 (90.6)	111 (86.7)	.13
Nationality (% non-Qatari)†	577 (84.6)	493 (89.0)	84 (65.6)	<0.001
Clinical				
**AACVPR Risk Category**				<0.001
Low	203 (29.7)	188 (33.9)	15 (11.7)	
Moderate	252 (37.0)	215 (38.8)	37 (28.9)	
High	227 (33.3)	151 (27.3)	37 (28.9)	
**Risk Factors**				
Tobacco use (% current)	175 (25.6)	107 (19.3)	68 (53.1)	<0.001
History of depression				.41
Not depressed	480 (70.4)	387 (69.9)	93 (72.7)	
Mild-moderate	74 (10.9)	58 (10.5)	16 (12.5)	
Severe	128 (18.7)	109 (19.7)	19 (14.8)	
Diabetes mellitus	277 (40.6)	224 (40.4)	53 (40.4)	.99
Hypertension	302 (44.3)	243 (43.9)	59 (46.1)	.65
**CR Indications**				
PCI	439 (64.4)	364 (65.7)	75 (58.6)	.13
Coronary artery disease	333 (48.8)	282 (50.9)	51 (39.8)	.02
Myocardial infarction	253 (37.1)	225 (40.6)	28 (21.9)	<0.001
CABG	147 (21.5)	122 (22.0)	25 (19.5)	.54
Valve replacement and disease	26 (3.8)	21 (3.8)	5 (3.9)	.95
Heart failure	25 (3.7)	16 (2.9)	9 (7.0)	.03
**Comorbidities / musculoskeletal issues**				
Back pain	26 (3.8)	9 (1.6)	17 (13.3)	<0.001
Musculoskeletal diseases	22 (3.3)	11 (2.0)	11 (8.6)	<0.001

*Difference between retention and lost to follow-up.
†49.7% East Asian, 35.7% Arab
AACVPR, American Association of Cardiovascular and Pulmonary Rehabilitation; CR, cardiac rehabilitation; PCI, percutaneous coronary intervention; CABG, coronary artery bypass grafting.
Note: n (%) or mean± standard deviation are shown.

References

1. Stone J JI, Lewanczuk R, et al. Canadian guidelines for cardiac rehabilitation and cardiovascular disease prevention: Translating knowledge into action 2009 [Available from: https://cacpr.wildapricot.org/Guidelines-for-Students.

2. American Association of Cardiovasular and Pulmonary Rehabilitation. Guidelines for cardiac rehabilitation and secondary prevention programs: Promoting health and preventing disease. 5th edition ed: Human Kinetics Publishers; 2013.

3. Ministry of Finance. Qatar State budget 2020 2020 [Available from: http://www.mof.gov.qa/en/Pages/StateBudget2020.aspx.

4. RAND health care. 36-Item short form survey (SF-36) 2019 [Available from: https://www.rand.org/health-care/surveys_tools/mos/36-item-short-form.html.

5. RAND health care. 36-Item short form survey (SF-36) scoring instructions 2019 [Available from: https://www.rand.org/health-care/surveys_tools/mos/36-item-short-form/scoring.html.

6. Brown K. A review to examine the use of SF-36 in cardiac rehabilitation. Br J Nurs. 2003;12(15):904-9.

7. Wise FM, Harris DW, Carter LM. Validation of the cardiac depression scale in a cardiac rehabilitation population. J Psychosom Res. 2006;60(2):177-83.

8. Kiropoulos LA, Meredith I, Tonkin A, Clarke D, Antonis P, Plunkett J. Psychometric properties of the cardiac depression scale in patients with coronary heart disease. BMC Psychiatry. 2012;12:216-.

9. Turk-Adawi K, Supervia M, Lopez-Jimenez F, Pesah E, Ding R, Britto RR, et al. Cardiac rehabilitation availability and density around the globe. EClinicalMedicine. 2019;13:31-45.

10. Grace SL, Turk-Adawi KI, Contractor A, Atrey A, Campbell N, Derman W, et al. Cardiac rehabilitation delivery model for low-resource settings. Heart (British Cardiac Society). 2016;102(18):1449-55.

11. Grace SL, Turk-Adawi KI, Contractor A, Atrey A, Campbell NR, Derman W, et al. Cardiac rehabilitation delivery model for low-resource settings: An international council of cardiovascular prevention and rehabilitation consensus statement. Prog Cardiovasc Dis. 2016;59(3):303-22.

12. Turk-Adawi K, Sarrafzadegan N, Fadhil I, Taubert K, Sadeghi M, Wenger NK, et al. Cardiovascular disease in the Eastern Mediterranean region: epidemiology and risk factor burden. Nature Reviews Cardiology. 2017;15:106.

13. Collins ZC, Suskin N, Aggarwal S, Grace SL. Cardiac rehabilitation wait times and relation to patient outcomes. Eur J Phys Rehabil Med. 2015;51(3):301-9.

14. The British Association for Cardiovascular Prevention and Rehabilitation. The BACPR standards and core components for cardiovascular disease prevention and rehabilitation 2017.(3rd Edition). 2017.
